# Adverse events: ART and the kidney: alterations in renal function and renal toxicity

**DOI:** 10.7448/IAS.17.4.19513

**Published:** 2014-11-02

**Authors:** Frank Post

**Affiliations:** Department of HIV/GU Medicine, King's College London, London, UK

## Abstract

Renal dysfunction is common in HIV-positive patients who receive antiretroviral therapy (ART). Several antiretrovirals have been associated with kidney disease progression, inhibition of renal tubular transporters that mediate creatinine secretion or impaired reabsorption of phosphate and low-molecular weight proteins. These aberrations of renal function are typically non-treatment limiting and of unclear clinical significance. By contrast, severe renal toxicity is infrequent in well-managed patents. Tenofovir-DF and atazanavir may cause acute tubular injury, tubule-interstitial nephritis or nephrolithiasis. Discontinuation of the offending drug is required to mitigate the adverse effects on kidney or bone. This presentation will discuss ART-associated changes in renal function and treatment-limiting renal toxicity in terms of incidence, risk factors, putative mechanism and provide recommendations for clinical practice.

